# Impact of sleep-related symptoms on clinical motor subtypes and disability in Parkinson’s disease: a multicentre cross-sectional study

**DOI:** 10.1136/jnnp-2017-316136

**Published:** 2017-08-28

**Authors:** Keisuke Suzuki, Yasuyuki Okuma, Tomoyuki Uchiyama, Masayuki Miyamoto, Ryuji Sakakibara, Yasushi Shimo, Nobutaka Hattori, Satoshi Kuwabara, Toshimasa Yamamoto, Yoshiaki Kaji, Shigeki Hirano, Taro Kadowaki, Koichi Hirata

**Affiliations:** 1 Department of Neurology, Dokkyo Medical University, Tochigi, Japan; 2 Department of Neurology, Juntendo University Shizuoka Hospital, Shizuoka, Japan; 3 Neuro-urology and Continence Center, Dokkyo Medical University Hospital, Tochigi, Japan; 4 Department of Clinical Medicine for Nursing, Dokkyo Medical University School of Nursing, Tochigi, Japan; 5 Department of Internal Medicine, Neurology Division, Sakura Medical Center, Toho University, Sakura, Japan; 6 Department of Neurology, Juntendo University School of Medicine, Tokyo, Japan; 7 Department of Neurology, Chiba University Graduate, School of Medicine, Chiba, Japan; 8 Department of Neurology, Saitama Medical University, Saitama, Japan

**Keywords:** Parkinson’s disease, sleep disorders, excessive daytime sleepiness, clinical motor phenotype

## Abstract

**Objectives:**

To investigate the impact of sleep disturbances on Parkinson’s disease (PD) clinical motor subtypes and disease-related disability in a multicentre setting.

**Methods:**

We report a cross-sectional relationship between sleep-related symptoms and clinical motor subtypes (tremor dominant (TD); intermediate; postural instability and gait disturbances (PIGDs)) identified in a multicentre study, including 436 patients with PD and 401 age-matched controls. PD-related sleep problems (PD-SP), excessive daytime sleepiness (EDS) and probable REM sleep behaviour disorder (pRBD) were evaluated using the PD sleep scale (PDSS)-2, Epworth Sleepiness Scale (ESS) and RBD screening questionnaire-Japanese version (RBDSQ-J), respectively.

**Results:**

PD-SP (PDSS-2 ≥18; 35.1% vs 7.0%), EDS (ESS ≥10; 37.8% vs 15.5%) and pRBD (RBDSQ-J ≥5; 35.1% vs 7.7%) were more common in patients with PD than in controls. The prevalence of restless legs syndrome did not differ between patients with PD and controls (3.4% vs 2.7%). After adjusting for age, sex, disease duration and Movement Disorder Society-Unified PD Rating Scale (MDS-UPDRS) part III score, the PIGD group had higher PDSS-2 and ESS scores than the TD group. The RBDSQ-J scores did not differ among the TD, intermediate and PIGD groups. A stepwise regression model predicting the MDS-UPDRS part II score identified the Hoehn and Yahr stage, followed by the number of sleep-related symptoms (PD-SP, EDS and pRBD), disease duration, MDS-UPDRS part III score, PIGD subtype, depression and MDS-UPDRS part IV score as significant predictors.

**Conclusion:**

Our study found a significant relationship between sleep disturbances and clinical motor subtypes. An increased number of sleep-related symptoms had an impact on disease-related disability.

## Introduction

Parkinson’s disease (PD) is a clinical entity that includes the following remarkably heterogeneous clinical presentations: tremor dominant, postural instability and gait difficulty (PIGD); several previous studies^1 2^ have suggested that early disease onset and rapid disease progression are also clinical presentations that may represent the underlying pathophysiological differences between individual patients.^3^ A recent review has related non-motor symptoms, such as sleep disturbances, olfactory disturbances and psychiatric symptoms, to specific clinical subtypes of PD, including the brainstem-dominant phenotype, limbic-dominant phenotype and cognitive-dominant phenotype.^4^ In PD, differences in the pathological progression patterns, such as bottom-up, top-down or the olfactory to limbic route, and in the severity of the Lewy-related pathology may have an impact on clinical presentations^5 6^; thus, clinical subtyping may be useful to indicate the underlying aetiology, prognosis or treatment response.

In PD, sleep disturbances and excessive daytime sleepiness (EDS) are multifactorial, complex problems attributed to disease pathology involving the brainstem/hypothalamus and are also secondarily due to motor/non-motor symptoms, as well as comorbid primary sleep disorders. Sleep disorders include restless legs syndrome (RLS) and REM sleep behaviour disorder (RBD); PD-related motor and non-motor symptoms can also cause nocturnal problems. These sleep problems are frequently observed and become prevalent in the late stage of the disease. In contrast, in addition to RBD, other sleep disorders and EDS can emerge in the premotor and early stages of PD, possibly reflecting the disease-related pathology responsible for sleep-wake disturbances.^7^ Because the pathophysiology of these sleep disorders involves both dopaminergic and non-dopaminergic mechanisms during the disease course, different types of sleep disorders may have different effects on the disease course, possibly constituting distinct phenotypes of PD. The relationship between RBD and the clinical subtypes akinetic-rigid and gait-freezing has been reported^8 9^; however, this finding remains controversial.^10 11^ Moreover, few studies have investigated the effect of non-motor symptoms on the clinical motor subtypes,^12^ and thus whether the presence of different non-motor symptoms can predict the specific clinical motor subtype remains an open question.

In this multicentre study, we aimed to characterise the clinical motor subtype related to the type and number of sleep-related symptoms in patients with PD and investigate the impact of sleep-related symptoms on disease-related disability.

## Patients and methods

We performed a multicentre study to assess the non-motor symptoms (NMS) of patients with PD between September 2014 and April 2016, including eight university hospitals in the Kanto region of Japan. The Kanto region consists of 7 prefectures, including the capital, Tokyo, and is called the Greater Tokyo Area, which has a population of approximately 42.6 million that accounts for one-third of the entire Japanese population, according to 2010 Population Census of Japan, Preliminary Counts of the Population and Households.

### Patients

We consecutively recruited 490 patients with PD (age 69.4±8.0 years; 225 males (M)) from outpatient clinics at the participating facilities. Bedridden patients or patients who were unable to answer the questionnaire were initially excluded from this study. After excluding dementia, which was defined as a Mini-Mental Status Examination (MMSE) score lower than 24, and missing data, 436 patients with PD (age 69.3±7.8 years; 197 M) were included in this study. Age-matched and sex-matched control subjects with no history of any neurological or psychiatric diseases (n=401; age 69.2±8.6 years; 187 M) were recruited from the medical staff and their friends and family.

### Methods

A diagnosis of PD was confirmed according to the UK Brain Bank Clinical Diagnostic Criteria,^13^ which defines the core motor features as bradykinesia and at least one of the following symptoms: rigidity, resting tremors or postural instability. All the patients were assessed by board-certified neurologists who were experienced in movement disorders; the patients also underwent brain imaging to exclude atypical parkinsonian syndrome or vascular parkinsonism. Drug-induced parkinsonism was excluded based on the clinical history. Disease severity was rated by Hoehn and Yahr (HY) staging. All the patients with PD completed the Japanese version of the MDS-UPDRS part II (motor experiences of daily living), MDS-UPDRS part III (motor examination) and MDS-UPDRS part IV (motor complications).^14^ Disease-related disability was assessed using the MDS-UPDRS part II.^15^ The clinical motor subtypes were defined using the MDS-UPDRS parts II and III, including the tremor-dominant subtype (ratio ≥1.5), PIGD subtype (ratio ≤1) and indeterminate subtype (ratios >1.0 and<1.5), as previously described.^16^ Early disease onset, defined as disease onset below 55 years of age, was also assessed.^2^

All the participants completed questionnaires regarding their habits, education and sleep status. The presence of visual hallucinations and falls during the past year were determined during interviews. The PD sleep scale (PDSS)-2, comprising 15 individual items for assessing nocturnal non-motor and motor problems, was used to evaluate PD-related sleep problems.^17^ The following three domain scores of the PDSS-2 were also evaluated: ‘disturbed sleep’ (items 1–3, 8 and 14); ‘motor symptoms at night’ (items 4–6, 12 and 13) and ‘PD symptoms at night’ (items 7, 9–11 and 15).^18^ PDSS-2 scores ≥18 were defined as clinically relevant PD-related sleep problems (PD-SP).^19^ The Japanese version of the Epworth Sleepiness Scale (ESS) was used, and excessive daytime sleepiness (EDS) was defined as an ESS score of 10 or greater.^20^ The RBD screening questionnaire-Japanese version (RBDSQ-J), was used, and an RBDSQ-J score of 5 or greater was defined as probable RBD (pRBD).^21^ RLS was diagnosed based on four essential features on the questionnaire after excluding RLS mimics such as positional discomfort, muscle cramp, venous stasis, vascular claudication and peripheral neuropathy and confirmed by the neurologists.^22^ The semi-structured Mini International Neuropsychiatric Interview based on the Diagnostic and Statistical Manual of Mental Disorders, Fourth Edition, was administered to the participants to assess depression (both minor and major depression).^23^ The levodopa equivalent dose (LED) was calculated based on the previously reported conversion factors.^24^

This study was conducted in accordance with the Declaration of Helsinki. The study was approved by the institutional review boards of the participating facilities, and written informed consent was obtained from all participants enrolled in the study.

### Statistical analysis

Mann-Whitney U test or Student’s t-test were used where appropriate to compare the continuous variables, and χ^2^ or Fisher’s exact tests were used to compare the categorical variables between the patients with PD and controls. The demographic characteristics of the tremor-dominant, intermediate and PIGD groups were compared using a one-way analysis of variance for continuous variables and χ^2^ test for categorical variables. When comparing the prevalence of sleep disorders among the patients with clinical subtypes of PD, differences were adjusted by age, sex, disease duration and MDS-UPDRS part III score using a binary logistic regression model. To predict PIGD, we performed a logistic regression analysis that included age, sex, disease duration, HY stage and LED as covariates. The correlation between MDS-UPDRS part II with other variables was analysed using Spearman’s rank correlation coefficients. A logistic regression analysis of PD-SP, EDS or pRBD as a dependent variable was performed, with age, sex, disease duration, HY stage, MDS-UPDRS parts II, III and IV, visual hallucinations, falls during the past year, depression, dopamine agonist use and levodopa use as the independent variables. A stepwise regression analysis was conducted to predict disease-related disability (MDS-UPDRS part II), which included age, sex, HY stage, disease duration, MMSE, MDS-UPDRS part III, MDS-UPDRS part IV, number of sleep-related symptoms (0–3; PDSS-2, pRBD or EDS), depression, tremor-dominant/PIGD score ratio, dopamine agonist use and levodopa use as the independent variables. Two-tailed p values of <0.05 were considered statistically significant. IBM SPSS software V.24.0 (IBM SPSS, Tokyo, Japan) was used for the statistical analyses, and GraphPad Prism for Windows (V.5.01; GraphPad Software, San Diego, California, USA) was used to generate the figure.

## Results

The mean onset age and disease duration of the patients with PD were 61.9±9.5 and 7.4±5.3 years, respectively. The mean HY stage was 2.3±0.7 (HY stage 1, n=41; HY stage 2, n=254; HY stage 3, n=120; HY stage 4, n=20; HY stage 5, n=1). The MDS-UPDRS part II, III and IV scores were 12.6±8.9, 28.6±13.3 and 2.0±3.3, respectively. Eighty-five patients (19.5%) had early disease onset. Thirty-four patients (7.8%) were drug naïve, and 227 (52.1%) and 365 (83.7%) patients were taking dopamine agonists or levodopa, respectively. Sixty (13.8%) patients were taking entacapone, 116 (26.6%) selegiline, 84 (19.3%) pramipexole, 57 (13.1%) ropinirole, 36 (8.3%) rotigotine, 35 (8.0%) pergolide, 10 (2.3%) cabergoline, 31 (7.1%) amantadine and 73 (16.7%) zonisamide. Eight patients (1.8%) were treated with deep brain stimulation. The demographic data of the patients with PD and the controls are shown in table 1. Compared with the controls, the patients with PD used less alcohol and tobacco. Regarding the sleep assessments, PD-SP, EDS and pRBD were significantly more common in the patients with PD than in the controls (table 1). However, the prevalence of RLS did not differ between the patients with PD and the controls (3.4% vs 2.7%, p=0.69).

**Table 1:** Demographic data and sleep-related symptoms in the patients with PD and controls

	PD	Controls	p Value
n (male/female)	436 (197/239)	401 (187/214)	0.67
Age (years)	69.3±7.8	69.2±8.6	0.38
Education (years)	12.5±2.8	12.2±2.8	0.10
Caffeine intake, n (%)	381 (87.4)	342 (85.3)	0.38
Caffeine (cup/day)	2.5±2.0	2.5±2.3	0.97
Alcohol, n (%)	196 (45.0)	217 (54.1)	0.0081
Smoking, n (%)	35 (8.0)	71 (17.7)	<0.0001
PDSS-2 total score	15.4±9.3	9.2±6.0	<0.0001
PD-related sleep problems, n (%)	153 (35.1)	28 (7.0)	<0.0001
ESS total score	8.4±5.2	6.1±3.5	<0.0001
EDS, n (%)	165 (37.8)	62 (15.5)	<0.0001
RBDSQ-J total score	3.9±2.8	1.6±1.8	<0.0001
pRBD, n (%)	153 (35.1)	31 (7.7)	<0.0001

ESS, Epworth Sleepiness Scale; RBDSQ-J, REM sleep behaviour screening questionnaire-Japanese version; EDS, excessive daytime sleepiness (ESS ≥10); PD, Parkinson’s disease; PD-SP, PD-related sleep problems (PDSS-2 ≥18); PDSS-2, PD sleep scale-2; pRBD, probable RBD (RBDSQ J ≥5).

The patients with PD were categorised into the following three groups according to their motor subtypes: tremor dominant (n=157, 36.0%), intermediate (n=45, 10.3%) and PIGD (n=234, 53.7%). The characteristics of the patients with PD according to the motor subtypes are shown in table 2. The PIGD phenotype was characterised by an older age, longer disease duration and higher scores on the MDS-UPDRS parts II, III and IV than the other subtypes. In the PIGD subtype, LED and the prevalence of hallucinations and falls during the past year were higher than in the other subtypes.

**Table 2:** Characteristics of the patients with PD classified by motor subtype

	Tremor dominant	Intermediate	PIGD	p Value
n (male/female)	157 (81/76)	45 (18/27)	234 (98/136)	0.13
Age (years)	68.1±7.2*	68.6±8.1	70.2±8.1	0.025
Disease duration (years)	5.7±4.3*	5.9±3.8*	8.7±5.8	<0.0001
Hoehn and Yahr stage, n (%)				
Stage 1	27 (17.2)*	7 (15.6)	7 (3.0)	
Stage 2	111 (70.7)*	28 (62.2)	115 (49.1)	
Stage 3	18 (11.5)*	10 (22.2)	92 (39.3)	
Stage 4	1 (0.6)*	0 (0.0)	19 (8.1)	
Stage 5	0 (0.0)*	0 (0.0)	1 (0.4)	
MDS-UPDRS part II	8.2±6.2*	8.8±7.3*	16.3±9.1	<0.0001
MDS-UPDRS part III	28.9±13.2	21.9±12*¶	29.6±13.3	0.016
MDS-UPDRS part IV	0.9±1.8*	1.4±2.4*	2.9±4.0	<0.0001
MMSE	28.2±2.0	28.3±1.9	27.8±2.0	0.14
Depression, n (%)	17 (10.8)	6 (13.3)	37 (15.8)	0.37
Initial presentation of motor symptoms, n (%)				<0.0001
Tremor	116 (73.9)*	19 (42.2)¶	72 (30.8)	
Bradykinesia	33 (21.0)*	22 (48.9)¶	146 (62.4)	
Tremor and bradykinesia	8 (5.1)*	4 (8.9)¶	16 (6.8)	
Early onset, n (%)	21 (13.4)*	6 (13.3)*	58 (24.8)	0.011
Falls during the past year, n (%)	26 (16.6)*	9 (20.0)*	101 (43.2)	<0.0001
Hallucinations, n (%)	6 (3.8)*	4 (8.9)	30 (12.8)	0.010
Wearing off, n (%)	32 (20.4)*	14 (31.1)	101 (43.2)	<0.0001
De novo, n (%)	16 (10.2)	4 (8.9)	14 (6.0)	0.30
LED (mg/day)	359.1±254.8*	415.8±283.8*	588.9±401.0	<0.0001
Dopamine agonist, n (%)	76 (48.4)	19 (42.2)	132 (56.4)	0.11
Levodopa, n (%)	120 (76.4)*	39 (86.7)	206 (88.0)	0.0082

*p<0.05 compared with the PIGD subtype; ¶p<0.05 compared with the tremor-dominant subtype.

LED, levodopa equivalent dose; MDS-UPDRS, Movement Disorder Society revision of the Unified Parkinson’s Disease Rating Scale; MMSE, Mini-Mental State Examination; PD, Parkinson’s disease; PIGD, postural instability and gait disturbance.

Table 3 shows the characteristics of the patients with PD according to the presence of sleep-related symptoms (PD-SP, EDS and pRBD). A mild male predominance was associated with EDS and pRBD. Compared with the patients without the above-mentioned sleep problems, the PD-SP and pRBD groups had longer disease durations, higher MDS-UPDRS part II, III and IV scores and LEDs and higher rates of depression, whereas the EDS group had higher MDS-UPDRS part II and IV scores and a higher rate of early disease onset. A specific clinical motor subtype, that is, an increased rate of PIGD was significantly related to the presence of PD-SP and EDS but not pRBD.

**Table 3:** Patients with PD classified by sleep-related symptoms

	PD-related sleep problems			Excessive daytime sleepiness			Probable RBD		
	Yes	No	p Value	Yes	No	p Value	Yes	No	p Value
n (male/female)	153 (76/77)	283 (121/162)	0.17	165 (91/74)	271 (106/165)	0.0011	153 (80/73)	283 (117/166)	0.028
Age (years)	70.1±7.3	68.8±8.1	0.11	68.7±8.4	69.6±7.5	0.25	69.1±7.4	69.3±8.1	0.76
Disease duration (years)	9.2±5.5	6.3±4.9	<0.0001	8.6±5.6	6.6±5.0	0.00010	8.5±5.9	6.7±4.9	0.0018
Hoehn and Yahr stage, n (%)			<0.0001			0.27			0.20
Stage 1	5 (3.3)	36 (12.7)		11 (6.7)	30 (11.1)		10 (6.5)	31 (11.0)	
Stage 2	78 (51.0)	176 (62.2)		99 (60.0)	155 (57.2)		91 (59.5)	163 (57.6)	
Stage 3	60 (39.2)	60 (21.2)		44 (26.7)	76 (28.0)		41 (26.8)	79 (27.9)	
Stage 4	9 (5.9)	11 (3.9)		10 (6.1)	10 (3.7)		10 (6.5)	10 (3.5)	
Stage 5	1 (0.7)	0 (0.0)		1 (0.6)	0 (0.0)		1 (0.7)	0 (0.0)	
MDS-UPDRS part II	17.8±9.0	9.8±7.5	<0.0001	15.9±9.6	10.6±7.9	<0.0001	15.2±9.5	11.2±8.3	<0.0001
MDS-UPDRS part III	31.5±13.1	26.9±13.1	0.00055	29.2±13.5	28.1±13.1	0.39	31.3±12.3	27.1±13.6	0.0016
MDS-UPDRS part IV	3.1±4.0	1.4±2.8	<0.0001	2.7±3.8	1.6±3.0	0.00096	2.4±3.4	1.8±3.3	0.047
MMSE	28.0±2.0	28.0±2.0	0.93	28.1±2.0	27.9±2.0	0.55	28.1±2.0	27.9±2.0	0.59
Depression, n (%)	38 (24.8)	22 (7.8)	<0.0001	27 (16.4)	33 (12.2)	0.22	29 (19.0)	31 (11.0)	0.021
Early onset, n (%)	32 (20.9)	53 (18.7)	0.58	43 (26.1)	42 (15.5)	0.0069	34 (22.2)	51 (18.0)	0.29
Clinical subtypes, n (%)			0.0014			0.00075			0.091
Tremor dominant	40 (26.1)	117 (41.3)		48 (29.1)	109 (40.2)		50 (32.7)	107 (37.8)	
Intermediate	13 (8.5)	32 (11.3)		10 (6.1)	35 (12.9)		11 (7.2)	34 (12.0)	
PIGD	100 (65.4)	134 (47.3)		107 (64.8)	127 (46.9)		92 (60.1)	142 (50.2)	
Fall during the past year, n (%)	63 (41.2)	73 (25.8)	0.00094	62 (37.6)	74 (27.3)	0.025	54 (35.3)	82 (29.0)	0.17
Hallucination, n (%)	25 (16.3)	15 (5.3)	0.00014	25 (15.2)	15 (5.5)	0.00074	25 (16.3)	15 (5.3)	0.00014
Wearing off, n (%)	74 (48.4)	73 (25.8)	<0.0001	70 (42.4)	77 (28.4)	0.0027	66 (43.1)	81 (28.6)	0.0022
De novo, n (%)	6 (3.9)	28 (9.9)	0.026	5 (3.0)	29 (10.7)	0.0030	10 (6.5)	24 (8.5)	0.47
LED (mg/day)	631.9±408.5	410.6±304.5	<0.0001	592.7±384.5	424.7±329.0	<0.0001	572.9±419.6	442.5±314.6	0.00089
Dopamine agonist, n (%)	87 (56.9)	140 (49.5)	0.14	94 (57.0)	133 (49.1)	0.11	85 (55.6)	142 (50.2)	0.28
Levodopa, n (%)	140 (91.5)	225 (79.5)	0.0012	148 (89.7)	217 (80.1)	0.0083	133 (86.9)	232 (82.0)	0.18

EDS, excessive daytime sleepiness (ESS ≥10); ESS, Epworth Sleepiness Scale; LED, levodopa equivalent dose; MDS-UPDRS, Movement Disorder Society revision of the Unified Parkinson’s Disease Rating Scale; MMSE, Mini-Mental State Examination; PD, Parkinson’s disease; PD-SP, PD-related sleep problems (PDSS-2 ≥18); PIGD, postural instability and gait disturbance; pRBD, probable RBD (RBDSQ-J ≥5); RBDSQ-J, REM sleep behaviour screening questionnaire-Japanese version.

**Figure 1:**
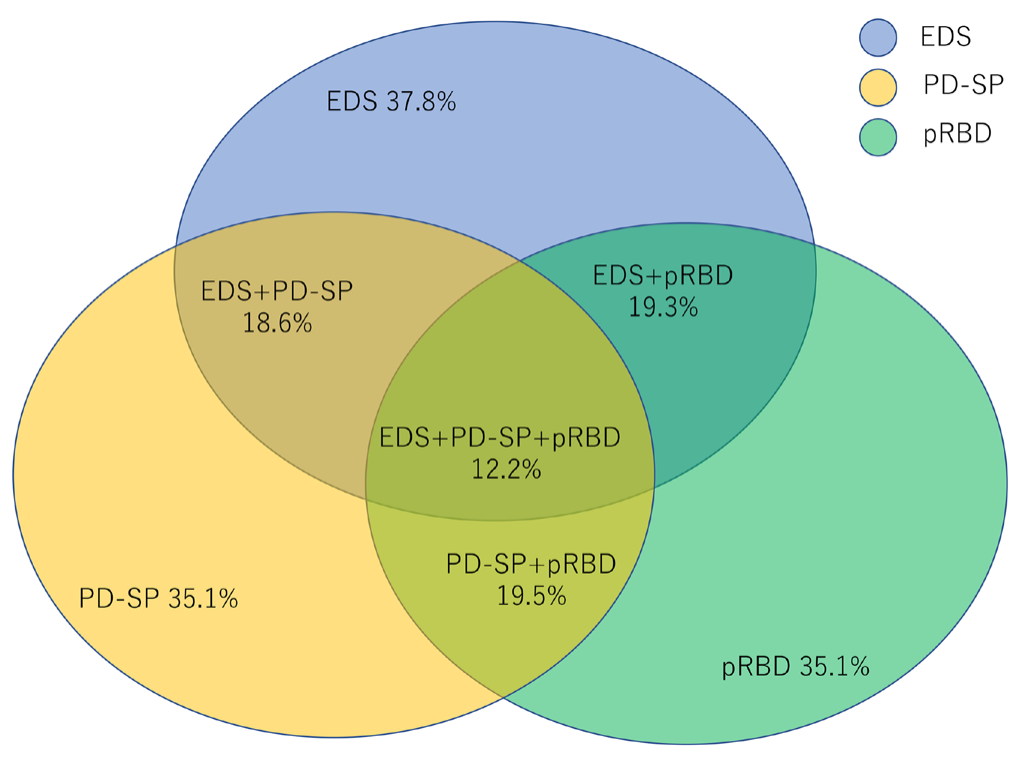
Prevalence and overlap of sleep-related symptoms in patients with PD. EDS, excessive daytime sleepiness; PD, Parkinson’s disease; PD-SP, PD- related sleep problems; pRBD, probable rapid eye movement sleep behaviour disorder.

There was a significant overlap of various sleep-related symptoms in the patients with PD. The prevalence of two out of three coexisting sleep-related symptoms (EDS, PD-SP and pRBD) was approximately 20%. The coexistence of all three symptoms (EDS, PD-SP and pRBD) was 12.2% (figure 1). The logistic regression analysis of EDS, pRBD and PD-SP using a likelihood ratio forward selection showed that male sex, the MDS-UPDRS part III score, hallucinations and the MDS-UPDRS part II score were contributing factors to EDS; male sex, hallucinations, the MDS-UPDRS part II score were contributing factors to pRBD and disease duration, depression and MDS-UPDRS part II score were contributing factors to PD-SP (table 4). Male sex (OR 1.821; 95% CI 1.135 to 2.921, p=0.013), HY stage (OR 3.818; 95% CI 2.460 to 5.926, p<0.0001), LED (OR 1.001; 95% CI 1.000 to 1.002, p=0.0014) and EDS (OR 1.8; 95% CI 1.130 to 3.039, p=0.014) were the significant predictors of the PIGD subtype in the logistic regression analysis.

**Table 4:** Logistic regression analysis of sleep-related symptoms

	OR	95% CI	p Value
(1) EDS			
Sex (male=1, female=2)	0.520	0.342 to 0.791	0.0022
MDS-UPDRS part III	0.982	0.964 to 0.999	0.045
Hallucination	2.075	1.001 to 4.304	0.049
MDS-UPDRS part II	1.083	1.053 to 1.113	<0.0001
(2) pRBD			
Sex (male=1, female=2)	0.663	0.441 to 0.998	0.049
Hallucination	2.570	1.264 to 5.222	0.0091
MDS-UPDRS part II	1.041	1.017 to 1.066	0.00082
(3) PD-SP			
Disease duration	1.050	1.003 to 1.099	0.035
Depression	2.917	1.553 to 5.480	0.00087
MDS-UPDRS part II	1.097	1.065 to 1.129	<0.0001

Independent variables: EDS, RBD or PD-SP plus age, sex, disease duration, Hoehn and Yahr stage, MDS-UPDRS parts II, III and IV, fall, visual hallucination, depression, dopamine agonist use, levodopa use.

A multivariate logistic model using a likelihood ratio forward selection.

EDS, excessive daytime sleepiness; MDS-UPDRS, Movement Disorder Society revision of the Unified Parkinson’s Disease Rating Scale; pRBD, probable RBD; PD-SP, PD-related sleep problems.

**Figure 2:**
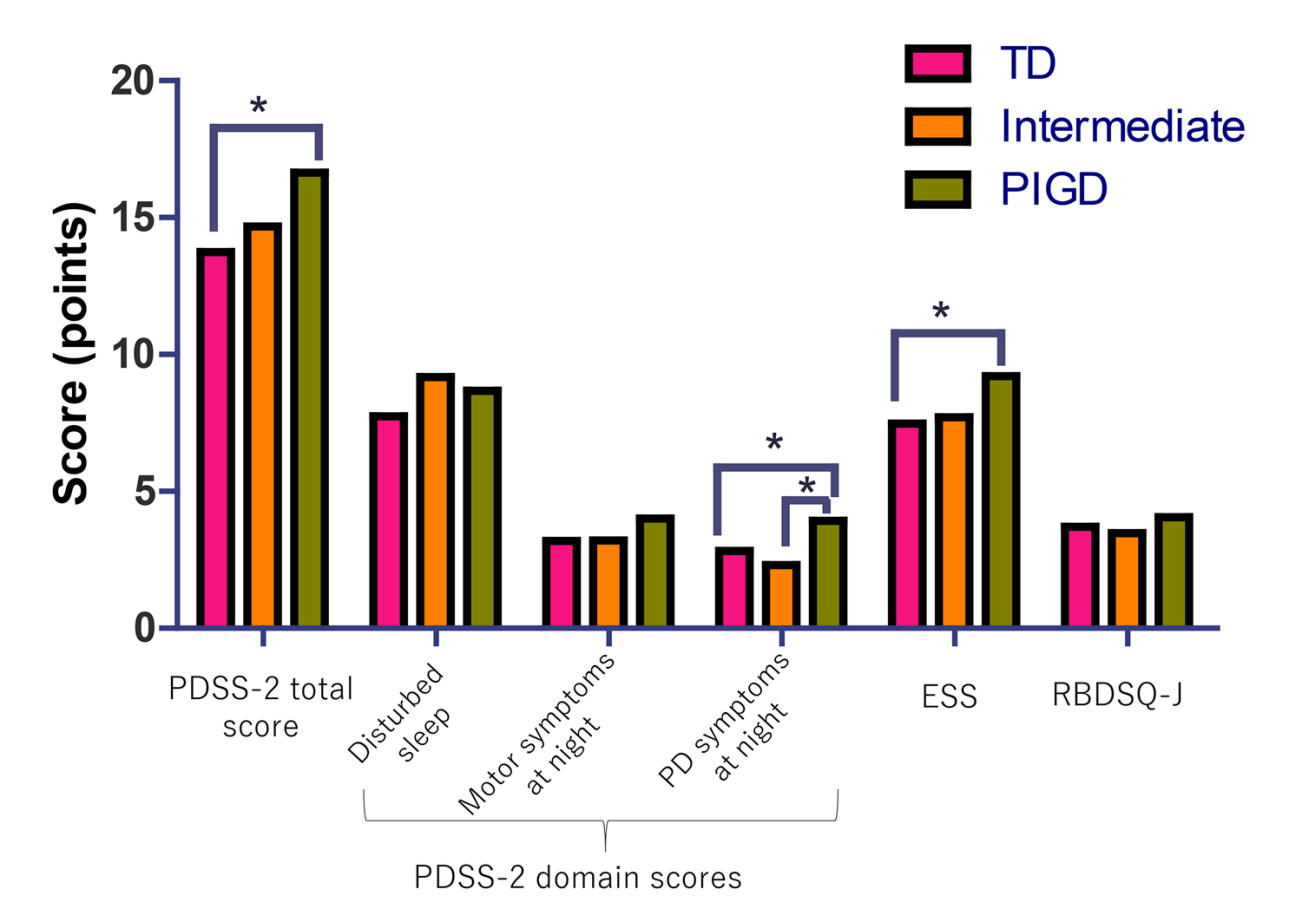
Clinical motor phenotype and sleep-related symptoms. *p<0.05, adjusted for age, sex, disease duration and MDS-UPDRS part III. PDSS-2=PD sleep scale-2; ESS=Epworth sleepiness scale; RBDSQ-J=REM sleep behaviour disorder screening questionnaire-Japanese version; TD=tremor dominant; PIGD=postural instability and gait disturbances. The three PDSS-2 domains include ‘disturbed sleep’, ‘motor symptoms at night’ and PD ‘symptoms at night’.

Figure 2 shows the comparison of the PDSS-2, ESS and RBDSQ-J scores among the tremor-dominant (TD), intermediate and PIGD groups. After adjusting for age, sex, disease duration and MDS-UPDRS part III, the PIGD group had a higher PDSS-2 total score than the tremor-dominant group and a higher PDSS-2 domain ‘PD symptoms at night’ score than the TD and intermediate groups. The ESS score was higher in the PIGD than in the TD group. The RBDSQ-J score did not significantly differ among the groups.

The MDS-UPDRS part II score was positively correlated with the disease duration (r=0.47, p<0.0001), HY stage (r=0.46, p<0.0001) and the MDS-UPDRS part III (r=0.42, p<0.0001), MDS-UPDRS part IV (r=0.34, p<0.0001), PDSS-2 (r=0.53, p<0.0001), ESS (r=0.36, p<0.0001) and RBDSQ-J (r=0.24, p<0.0001) scores. The MDS-UPDRS part II score was negatively correlated with the TD/PIGD ratio (r=−0.38, p<0.0001). A significant correlation was also obtained with the PDSS-2 three domain scores: ‘disturbed sleep’ (r=0.42, p<0.0001), ‘motor symptoms at night’ (r=0.44, p<0.0001) and ‘PD symptoms at night’ (r=0.51, p<0.0001).

To determine the predictors for disease-related disability in the patients with PD, we performed a stepwise regression analysis. In the stepwise regression model of disease-related disability (MDS-UPDRS part II), the final model showed that the HY stage, followed by an increased number of sleep-related symptoms (0–3; PD-SP, EDS and pRBD), disease duration, MDS-UPDRS part III score, PIGD subtype, depression and MDS-UPDRS part IV score were significant predictors (table 5).

**Table 5:** Stepwise linear regression analysis of the MDS-UPDRS  part II

	Standardised regression coefficient	SE	p Value
HY stage	0.143	0.556	0.0011
Number of sleep-related symptoms	0.226	0.293	<0.0001
Disease duration	0.193	0.073	<0.0001
MDS-UPDRS part III	0.264	0.028	<0.0001
TD/PIGD ratio	−0.208	0.162	<0.0001
Depression	0.117	0.920	0.0012
MDS-UPDRS part IV	0.109	0.108	0.0085

Final model, adjusted R^2^=0.539.

Dependent variables; age, sex, HY stage, disease duration, MMSE, MDS-UPDRS III, IV, number of sleep-related symptoms (0–3; EDS, PD-SP and pRBD), depression and TD/PIGD ratio.

HY, Hoehn and Yahr stage; MDS-UPDRS, Movement Disorder Society revision of the Unified Parkinson’s Disease Rating Scale; TD, tremor dominant; PIGD, postural instability and gait disturbance.

## Discussion

In this cross-sectional multicentre study, we confirmed a significantly higher prevalence of sleep-related symptoms, such as PD-SP, EDS and pRBD, in the patients with PD than in the age-matched and sex-matched controls; these results are similar to those of previous cross-sectional studies.^25–28^ Prospective studies have also shown that the prevalence of EDS increases during the disease course^29–31^; however, the prevalence of pRBD^32 33^ or insomnia has been observed to fluctuate^34 35^ during follow-ups. Gjerstad *et al* 34 have reported that the prevalence of insomnia in patients with PD fluctuates from 54% to 60% over an 8-year period; meanwhile in a 5-year-prospective study, the prevalence of insomnia was 27% at baseline, and among the patients without insomnia at baseline, 33% developed insomnia at some point during the follow-up.^35^ In addition, we found a significant overlap in three sleep-related symptoms (EDS, PD-SP and pRBD). The prevalence of two and three coexisting sleep-related symptoms was one-fifth and one-eighth, respectively (figure 1). In our study, the prevalence of RLS was low in our total cohort and was similar between the patients with PD and controls (3.4% vs 2.7%), although an increased prevalence of RLS has been reported in Japanese patients with PD compared with that in controls (12% vs 2.3%).^36^

When categorising the patients with PD based on the presence or absence of specific sleep problems (PD-SP, EDS or pRBD), we found relationships between the sleep-related symptoms and specific clinical parameters. Compared with the patients with PD without sleep-related symptoms, the PD-SP and pRBD groups had longer disease durations, higher MDS-UPDRD part II, III and IV scores and greater rates of depression, while the EDS group had higher MDS-UPDRS part II and IV scores and a higher rate of early disease onset. These findings suggest that motor impairments and depression are associated with PD-SP and pRBD. Notably, we found a significant link between the clinical motor subtypes and sleep-related symptoms (PD-SP, EDS and pRBD) by observing that (1) the PIGD subtype was the predominant clinical motor subtype in the PD-SP and EDS groups but not in the pRBD group (table 3) and (2) the PIGD group had significantly higher PDSS-2 and ESS scores than the TD group after correcting for age, sex, disease duration and MDS-UPDRS III (figure 2). Our study is the first attempt to cross-sectionally correlate the clinical motor subtypes and several sleep-related symptoms, such as PD-SP, EDS and pRBD, in patients with PD. Although, in our study, the ESS and PDSS-2 total scores remained higher in the PIGD group than in the TD groups in the general linear model after controlling for the confounding factors, because the PIGD subtype showed severe motor symptoms and disease severity compared with the TD phenotype, the disease progression might have influenced PD-SP and EDS. Furthermore, pRBD was not associated with a specific motor subtype.

We also determined the clinical contributors to disease-related disability, as measured by the MDS-UPDRS part II, which is the recommend tool for disability assessment.^15^ Disease-related disability was significantly correlated with disease severity, disease duration and the MDS-UPDRS part III and IV scores, as well as the sleep-related symptoms, such as PD-SP, pRBD and EDS. In a regression model, in addition to disease severity, an increased number of sleep-related symptoms (PD-SP, EDS and pRBD), the PIGD subtype, motor function, motor complications and depression had a significant impact on disease-related disability. Axial impairment, mood, depression and cognitive impairment have been previously reported as determinants of disability and impaired quality of life in patients with PD.^37 38^ PD-SP, as evaluated by the PDSS-2, has been correlated with quality of life^17 18^; however, this association has not been assessed in a large sample of patients with PD. Although Neikrug *et al* 39 have reported that an increasing number of sleep disorders, such as RLS and RBD, is associated with increased non-motor symptoms. Importantly, our finding demonstrating that an increased number of sleep-related symptoms (PD-SP, EDS and pRBD) had a significant impact on disease-related disability has never been reported. Altogether, these observations may imply that these sleep disorders and related symptoms occur in association with the degree and distribution of the degeneration of brain dopaminergic/non-dopaminergic neurons, which could determine certain motor and non-motor phenotypes of PD.

A major limitation of this study is the lack of polysomnography to assess the sleep status of the patients. In our study, the incidence of RLS was much lower than that previously reported and RLS had less of an impact on disability than other sleep-related symptoms, such as PD-SP, EDS and pRBD. One possible explanation is that the range of the severity of RLS in the patients may have influenced the results. To characterise RLS in patients with PD, further studies analysing RLS and leg motor restlessness, which is reported to be more common in untreated patients with PD than in controls,^40^ are necessary. As another limitation, our study is a cross-sectional evaluation that included nearly an entire cohort of patients treated with dopaminergic drugs. Although we excluded dementia, defined as an MMSE score <24, we did not assess mild cognitive impairment or impulse control disorder, which may affect sleep problems, RBD and EDS. The possible effect of the use of different dopamine agonists on sleep-related symptoms was also not assessed in this study. One could argue that because the motor phenotype can change during the disease course of PD, a different motor subtype may represent only the stage of the disease. Nevertheless, our preliminary approach to correlating sleep-related symptoms with the clinical motor subtypes of a large sample of nearly all patients with PD treated with dopaminergic drugs in a multicentre study, which is close to ‘a real-world clinical setting’, is potentially meaningful. Further prospective studies are necessary to assess the relationship between clinical motor subtypes and sleep disorders and related symptoms in PD.

In conclusion, our cross-sectional survey results indicate a possible relationship between the clinical motor subtypes of PD and sleep-related symptoms and demonstrate the importance of the clinical assessment and management of sleep-related symptoms (PD-SP, EDS and pRBD), which have a significant impact on disease-related disability compared with motor symptoms in patients with PD.
